# Radiation therapy margin reduction for patients with localized prostate cancer: A prospective study of the dosimetric impact and quality of life

**DOI:** 10.1002/acm2.14198

**Published:** 2023-11-12

**Authors:** Akila Kumarasiri, Indrin J. Chetty, Suneetha Devpura, Deepak Pradhan, Ibrahim Aref, Mohamed A. Elshaikh, Benjamin Movsas

**Affiliations:** ^1^ Department of Radiation Oncology Henry Ford Health Detroit Michigan USA

**Keywords:** deformable dose accumulation, margin reduction, patient reported outcomes (PROs), quality of life (QOL)

## Abstract

**Objectives:**

To investigate the impact of reducing Clinical Target Volume (CTV) to Planning Target Volume (PTV) margins on delivered radiation therapy (RT) dose and patient reported quality‐of‐life (QOL) for patients with localized prostate cancer.

**Methods:**

Twenty patients were included in a single institution IRB‐approved prospective study. Nine were planned with reduced margins (4 mm at prostate/rectum interface, 5 mm elsewhere), and 11 with standard margins (6/10 mm). Cumulative delivered dose was calculated using deformable dose accumulation. Each daily CBCT dataset was deformed to the planning CT (pCT), dose was computed, and accumulated on the resampled pCT using a parameter‐optimized, B‐spline algorithm (Elastix, ITK/VTK). EPIC‐26 patient reported QOL was prospectively collected pre‐treatment, post‐treatment, and at 2‐, 6‐, 12‐, 18‐, 24‐, 36‐, 48‐, and 60‐month follow‐ups. Post ‐RT QOL scores were baseline corrected and standardized to a [0–100] scale using EPIC‐26 methodology. Correlations between QOL scores and dosimetric parameters were investigated, and the overall QOL differences between the two groups (QOL_Margin‐reduced_‐QOL_control_) were calculated.

**Results:**

The median QOL follow‐up length for the 20 patients was 48 months. Difference between delivered dose and planned dose did not reach statistical significance (*p* > 0.1) for both targets and organs at risk between the two groups. At 4 years post‐RT, standardized mean QOL_Margin‐reduced_‐QOL_control_ were improved for *Urinary Incontinence, Urinary Irritative/Obstructive, Bowel*, and *Sexual* EPIC domains by 3.5, 14.8, 10.2, and 16.1, respectively (higher values better). The control group showed larger PTV/rectum and PTV/bladder intersection volumes (7.2 ± 5.8, 18.2 ± 8.1 cc) than the margin‐reduced group (2.6 ± 1.8, 12.5 ± 8.3 cc), though the dose to these intersection volumes did not reach statistical significance (*p* > 0.1) between the groups. PTV/rectum intersection volume showed a moderate correlation (*r* = −0.56, *p* < 0.05) to *Bowel* EPIC domain.

**Conclusions:**

Results of this prospective study showed that margin‐reduced group exhibited clinically meaningful improvement of QOL without compromising the target dose coverage.

## 1 INTRODUCTION

Recent advances in image guided intensity modulated radiation therapy allow the delivery of highly conformal dose distributions for treating localized prostate cancer patients, while minimizing the dose delivered to organs at risk (OAR) such as rectum, bladder, bowel, and femoral heads.^1^ Dose to bladder and rectum is often affected by the treatment margins added to the clinical target volume (CTV) to create the planning target volume (PTV), to account for setup uncertainties and inter‐ and intra‐fraction motion of the prostate.^2^ PTV margins of 10 mm overall with 6 mm at the prostate/rectum interface has been a standard historical approach for conventional fractionation.^3^ Multiple retrospective dosimetric modeling studies have suggested that reducing this margin may lead to reduced OAR toxicity.^4^, ^5^, ^6^ A prospective clinical trial has also shown evidence that reduced planning margins achievable using real‐time Calypso‐based tracking produced a benefit in reduced morbidity.^7^ However, this strategy requires the placement of an invasive fiducial marker.

As PTV margins are reduced, loss of target coverage and dose deviations to OAR from planned dose due to setup uncertainties and anatomical variations may become more prominent. Therefore, reduced margins require employing stringent strategies such as daily image guided localization, to ensure there is no potential loss of target coverage.^3^, ^8^ Fiducial markers implanted in the prostate such as calypso and/or daily cone beam CT (CBCT) imaging can be used for motion management and daily localization, while a cumulative dose‐based approach can be utilized to estimate the actual delivered dose to targets and OAR.^9^, ^10^ Moreover, toxicity of rectum and bladder, and patient quality of life (QOL) post radiotherapy, need to be assessed.

In a prospective clinical trial, we are investigating the impact of reducing CTV‐to‐PTV margin to 5 mm uniform except for 4 mm at the prostate/rectum interface. In this paper, we report the deformable dose accumulation and QOL results of 20 patients enrolled in this trial (including 11 control patients who were treated with standard margin of 10 mm uniformly except for 6 mm at the prostate/rectum interface).

## 2 METHODS

### 2.1 Dataset

The patient cohort consisted of 20 patients who were enrolled in a prospective Institutional Review Board approved clinical trial. This includes nine reduced‐margin patients (4 mm at prostate/rectum interface, 4 mm elsewhere), and 11 control patients with standard margins (6 mm at the prostate/rectum interface, 10 mm elsewhere). Patient eligibility criteria included histologically confirmed prostate adenocarcinoma with stages T1b‐T2b, Gleason score 6−7, and prostate‐specific antigen (PSA) < 15 ng/mL. Patients with evidence of distant metastases, regional lymph node involvement, radical prostatectomy, or previous pelvic irradiation were excluded. All patients were treated with radiotherapy alone.

The median [range] of age, Gleason scores, and PSA levels of the two arms of the study are shown in Table 1. Differences in these parameters between the two groups are not statistically significant (*p* > 0.2 for all parameters).

**TABLE 1:** Patient characteristics.

	Age (years)	Gleason score	PSA (ng/mL)	Stage
Reduced‐Margin (*N* = 9)	60 [51–82]	7 [6–7]	9.8 [1.8–12.2]	All T1c
Standard Margin (*N* = 11)	67 [60–70]	7 [6–7]	5.1 [2–13.1]	10 T1c, 1 T2a

*Note*: Age, Gleason score, PSA levels (median [range]), and cancer staging.

### 2.2 Planning and treatment

Patients were simulated and treated with full/partially full bladder and empty rectum, in a supine head‐first position. For bladder and rectum preparation, patients were advised to have a bowel movement prior to leaving home, and drink 16 oz of water approximately 30 min prior to simulation and each treatment. The CTV, PTV, and OAR were contoured by the attending radiation oncologist. A nominal dose of 79.2 Gy to the PTV in 44 fractions (1.8 Gy/fraction) was planned with a single or 2‐arc VMAT technique. OARs contoured included bladder, rectum, femoral heads, and penile bulb. Bladder was contoured from its base to the dome, and the rectum from the anus (at the level of the ischial tuberosities) for a length of 15 cm or up to where the rectosigmoid flexure is identified. RTOG 0126 toxicity criteria for rectum, bladder, and penile bulb were used as planning constraints for both arms of the study^11^ (Table 2). Minimum dose to PTV was kept >95% of the prescription dose, while maximum was kept <107%.

**TABLE 2:** Organs at risk constraints used for treatment planning.

Organ at risk	No more than 15% volume receives dose that exceeds	No more than 25% volume receives dose that exceeds	No more than 35% volume receives dose that exceeds	No more than 50% volume receives dose that exceeds
Bladder	80 Gy	75 Gy	70 Gy	65 Gy
Rectum	75 Gy	70 Gy	65 Gy	60 Gy
Penile bulb	Mean dose less than or equal to 52.5 Gy			
Femoral head	Less than or equal to 10% of each femoral head should receive ≥ 50 Gy			

For daily image guided localization, a pelvis mode CBCT scan (half fan full rotation) was acquired immediately prior to each treatment. Bladder and rectum filling was assessed and remediated prior to treatment for cases of excessive rectal filling/gas or empty bladder. An online auto fusion (three translational degrees of freedom) was performed for bony alignment, followed by manual fine tuning to match the soft tissue in the vicinity of the PTV. Patients were planned with Aria/Eclipse Treatment Planning System (Varian Medical Systems/Siemens Healthineers), and treated using Varian TrueBeam or Trilogy class machines equipped with Varian Millennium 120 MLCs.

### 2.3 Dose accumulation

To mitigate known dose calculation uncertainties associated with CBCT images such as increased noise level and imaging artifacts,^12^ deformably resampled CT images were used for the *dose of the day* calculations. To obtain resampled CT images, the planning CT of each patient was registered to daily CBCTs using a parameter optimized Elastix B‐spline deformable image registration (DIR) algorithm.^13^ The patient treatment plan was then mapped to the resampled CT images using online registrations that were originally used for daily image guidance. Then dose was calculated using the AAA algorithm (v11). The calculated daily doses were then transferred and accumulated on the corresponding planning CT images using the B‐spline based Elastix transformation and energy‐mass mapping.^14^ Validation of this DIR‐based dose accumulation process including DIR parameter optimization and are available elsewhere.^9^, ^10^

### 2.4 QOL data collection and analysis

Patient reported QOL data were collected using EPIC‐26 based questionnaires pre‐treatment, post‐treatment, and at 2‐, 6‐, 12‐, 18‐, 24‐, 36‐, 48‐, and 60‐month follow‐up time points. EPIC‐26 methodology^15^ was then used to standardize each patient response item to a [0–100] scale. All post‐treatment QOL scores were baseline corrected by subtracting the corresponding pre‐treatment QOL score, and mean scores were then calculated for each time point. Finally, a baseline‐corrected EPIC domain summary of mean QOL scores for each collection time point was calculated for EPIC domains categorized as *Urinary Incontinence*, *Urinary Irritative/Obstructive*, *Bowel, Sexual*, and *Hormonal*, by using the item groupings for each domain using EPIC methodology.

To compare post treatment QOL scores between the two arms, QOL differences between the margin reduced group and control group (QOL_Margin‐reduced_‐QOL_control_) were calculated for each post‐treatment follow up time point. To assess if the differences in QOL between the two groups are clinically meaningful, Minimally Important Difference (MID) methodology validated by Skolarus et al.^16^ was used.

Correlations of post‐RT QOL to OAR dosimetric parameters were also investigated. Delivered and planned D_mean_ and D_max_ to OAR, as well as hot spots in OAR (PTV/bladder and PTV/rectum intersection volumes that is getting the full prescription dose) were correlated to relevant EPIC domains using Pearson correlation statistics with a 2‐sided significance level of 0.05. The Student's *t*‐test was used to determine statistical significance (*p* < 0.05) of dosimetric parameters and QOL correlations. Statistical analysis was performed using IBM SPSS v26 (IBM, Armonk, New York).

## 3 RESULTS

Age, Gleason score, and PSA level differences between the two groups (Table 1) were not significantly different pre‐RT (*p* > 0.2). In addition, planned PTV D95 and V95 for margin reduced and standard margin groups were (78.7 ± 1.0 Gy, 99.67 ± 0.33%) and (78.5 ± 1.4 Gy, 99.67 ± 0.33%), respectively. Differences of D95 and V95 between the two groups were statistically insignificant (*p* > 0.6, *p* > 0.1, respectively), suggesting planned target coverage is similar between the two groups.

Post‐RT, the margin reduced group had a 100% PSA control rate at the most recent follow up, while the control group had a 73% PSA control rate where three out of the 11 patients had long term PSA failure. Nadir PSA + 2 ng/mL was used as the definition of biochemical failure.^17^

**FIGURE 1:**
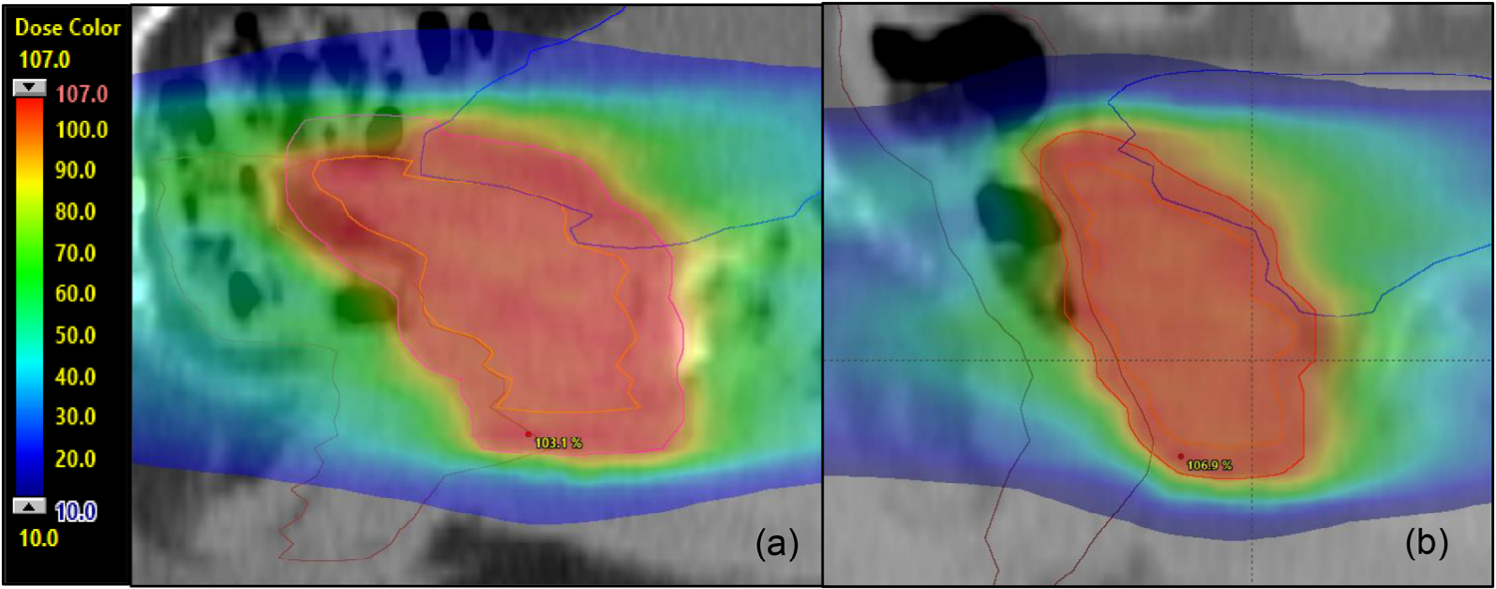
Sagittal view of PTV (red), CTV (orange), rectum (brown), and bladder (blue) of a typical patient from (a) standard margin group (b) reduced margin group, at planning isocenter (typically set to center of prostate). The planned dose distribution is shown.

Figure 1 shows an example comparison of dose distribution between the two groups. Sagittal view at the center of PTV, along with rectum and bladder contours are presented of a typical patient from (a) standard margin group (b) reduced margin group with normalized dose. Due to the larger PTV margins used, a larger volume of the bladder and rectum of the standard margin patient receives the prescription dose.

Table 3 shows the delivered (cumulative) mean dose (D_mean_) to PTV and OARs, estimated by deformably accumulating *dose of the day* as described in methods section. The planned mean dose, and the difference between the two (i.e., difference from planned dose in Gray and as a percentage) is also shown. For the PTV and the organs considered, differences in dose deviations from planned dose between the margin reduced and standard margin groups were not statistically significant (*p* > 0.1). Differences between delivered and planned maximum dose (D_max_, not presented) are also not statistically significant (*p* > 0.2). This suggests that lowering the margin from 10/6 mm to 5/4 mm does not lead to a significant loss of target coverage or significant changes in dose to OAR from planned dose, with daily CBCT localization.

**TABLE 3:** D_mean_ delivered (i.e., cumulative) and planned in Gy, for reduced margin patients (*N* = 9) and standard margin patients (*N* = 11).

	Reduced margin			Standard margin		
Target/OAR	D_mean_ delivered	D_mean_ planned	Deviation in Gy (and as a %)	D_mean_ delivered	D_mean_ planned	Deviation in Gy (and as a %)
PTV	78.0 ± 1.3	80.2 ± 0.6	−2.2 ± 1.0(−2.8 ± 1.3%)	76.9 ± 2.9	77.7 ± 2.9	−0.8 ± 2.0(−1.0 ± 2.6%)
Prostate	80.9 ± 0.4	80.6 ± 0.4	0.3 ± 0.5(0.4 ± 0.6%)	79.8 ± 1.8	79.9 ± 1.6	0.9 ± 1.4(1.1 ± 1.8%)
Bladder	33.3 ± 9.5	34.1 ± 10.2	−0.7 ± 2.6(−1.5 ± 6.7%)	31.9 ± 12.0	32.5 ± 17.8	−0.7 ± 3.1(−1.2 ± 10.4%)
Rectum	33.0 ± 4.3	35.1 ± 3.6	−2.1 ± 1.3(−6.2 ± 3.9%)	38.7 ± 5.9	39.7 ± 5.1	−1.0 ± 2.4(−2.6 ± 6.2%)
Seminal Vesicles	74.5 ± 9.0	78.1 ± 5.6	−3.6 ± 3.6(−5.0 ± 5.5%)	75.2 ± 4.4	76.6 ± 3.0	−1.4 ± 2(−1.9 ± 2.9%).

*Note*: The deviation is shown in Gy and as a % (within parenthesis).

The median follow‐up length for the first 20 patients in this trial was 48 months. Table 4 presents the difference in standardized and baseline corrected EPIC QOL values between the two groups (QOL_Margin‐reduced_ ‐QOL_control_). Value for each time point [end of RT – 48 months], as well as an overall mean QOL difference are shown for the five EPIC‐26 domains. The number of patients reported at each timepoint, and the compliance rate (defined as number of QOL responses received/number of QOL responses sent out for that time point) is also indicated.

**TABLE 4:** Baseline corrected QOL_Margin‐reduced_—QOL_control_ at each time point, and overall mean values, for each EPIC domain.

Time point	Urinary incontinence	Urinary irritative/obstructive	Bowel	Sexual	Hormonal
End of RT (*N* = 20, 100%)	−6.4	13.4	6.3	6.6	−2.6
2 month post RT (*N* = 20, 100%)	3.1	8.2	6.6	8.3	−3.2
6 month post RT (*N* = 19, 95%)	−2.0	8.5	19.0	14.9	4.4
12 month post RT (*N* = 19, 95%)	5.5	10.3	8.0	19.9	7.1
24 month post RT (*N* = 17, 85%)	−1.5	9.5	15.6	14.6	−13.0
36 month post RT (*N* = 16, 80%)	5.6	16.5	9.0	20.2	−13.8
48 month post RT (*N* = 13, 72%)	−3.9	6.3	4.9	6.2	−14.2
Mean QOL_Margin‐reduced—_QOL_control_	3.5	14.8	10.2	16.1	−4.9
Minimally important difference range [Skolarus et al. Urology 2015]	6–9	5–7	4–6	10–12	4–6

*Note*: Number of patients at each time point (*N*), and compliance rate (% of patients who responded to the QOL questionnaires), are indicated within parenthesis.

To determine the clinical significance in the change of QOL, the difference in mean QOL scores between the two groups for the [end of RT – 48 months] period was compared against the minimally important difference range.^16^ Mean QOL_Margin‐reduced_ ‐QOL_control_ for domains Urinary Irritative/Obstructive, Bowel, and Sexual are above the minimally important difference, suggesting that QOL increase in margin–reduced patients are clinically meaningful for these three domains.

Following statistical correlations between dose to OAR and EPIC QOL domains were observed for average QOL over [end of RT – 48 months]; Urinary Incontinence domain showed statistically significant (*p* < 0.05) moderate correlations to Bladder D_mean_ (*r* = −0.57), V_80_ (*r* = −0.46), and V_50_ (*r* = −0.47). Bladder dose correlations with Urinary Irritative/Obstructive domain, and rectum dose correlations with Bowel domain were all statistically insignificant (*p* > 0.05).

Following statistically significant (*p* < 0.05) correlations were observed for QOL at each timepoint; Urinary Incontinence domain moderate correlations to Bladder V80 at 6 months (*r* = −0.53), Urinary Irritative/Obstructive domain moderate correlation to Bladder V70 at 36 months (*r* = −0.6) Bowel domain moderate correlation to Rectum V70 at 2 months (*r* = −0.6), Rectum V50 at 2 months (*r* = −0.54), and Rectum V70 at 6 months (*r* = −0.49)

We further investigated delivered dose distributions by evaluating the PTV/bladder, and PTV/rectum intersection volumes (i.e., *hot spot* volumes that are getting close to the full prescription dose). Table 5 lists the average volumes and the dosimetric indices. Although the D_max_, D_mean_, and D_min_ values are not statistically different between the groups (*p* > 0.1), the standard margin group has significantly larger PTV/bladder and PTV/rectum intersection volumes (*p* < 0.05). This suggests that the hot spots in the bladder and rectum are significantly smaller for the margin‐reduced group. The PTV/rectum intersection volume shows a moderate correlation to the Bowel EPIC domain (Pearson's coefficient *r* = −0.56, *p* < 0.05). We did not observe a statistically significant correlation between dose parameters of the bladder and bladder related EPIC domains.

**TABLE 5:** Mean ± Std. Dev. intersection volumes (in cc) and dose metrics (in Gy) for the two groups.

Target/OAR	Reduced margin (5/4 mm, *N* = 9)				Standard margin (10/6 mm, *N* = 11)			
	Volume	D_min_	D_max_	D_mean_	Volume	D_min_	D_max_	D_mean_
PTV/Bladder intersection	12.5 ± 8.3	70.7 ± 9.9	83.8 ± 1.5	79.4 ± 2.6	18.2 ± 8.1	70.3 ± 8.3	83.9 ± 1.2	80.4 ± 1.7
PTV/Rectum intersection	2.6 ± 1.8	70.1 ± 11.9	83.5 ± 2.2	79.3 ± 1.6	7.2 ± 5.8	70.4 ± 8.1	83.1 ± 2.3	79.7 ± 3.2

## 4 DISCUSSION

Decreasing dose to bladder and rectum by reducing CTV‐to‐PTV margins for prostate RT using daily image guidance has been well‐studied, though mostly retrospectively. For example, Maund et al.^6^ retrospectively found that when daily online CBCT image guidance is used, margins of 3−4 mm is achievable without compromising tumor control. They theorized through NTCP modeling that sparing of surrounding normal tissue could potentially lead to a reduction in rectal toxicity. Hammoud et al.^3^ also retrospectively investigated the dosimetric effect on reducing PTV margins from 10/6 mm to 5/3 mm, and found that reducing margin allowed a 30%−50% sparing of bladder and rectal high‐dose regions. Moreover, multiple retrospective dosimetric modeling studies have shown that reducing the margin could potentially lead to reduced OAR toxicity.^4^, ^5^, ^6^

However, as PTV margins are reduced, loss of target coverage due to setup uncertainties and inter‐ and intra‐fraction motion could potentially be an issue. Mayyas et al.^2^ found that when daily CBCT‐based localization is used, the setup margin can be reduced to 3–5 mm to account for setup uncertainties. A study by Gill et al.^18^ demonstrated using rigid registration, that when daily CBCT localization was used, a 3 mm PTV margin allowed for CTV to be covered for 99% of cases. However, due to inter fraction deformations in the prostate and surrounding soft tissue, a DIR‐based dose accumulation workflow may lead to a better estimate of actual delivered dose. A retrospective dose accumulation study by Wen et al.^19^ demonstrated that margin reduction could effectively be used to improve the therapeutic ratio, but has to be used with caution due to large inter patient variability. They concluded that a prospective study with a large patient population is warranted.

Studies have also investigated the effect of PTV margin reduction on toxicity, with somewhat mixed results. A prospective clinical trial by Sandler et al. has investigated the impact of margin reduction on rectum toxicity^7^ using patient reported QOL. By using real‐time tracking of prostate during radiotherapy using Calypso markers, they were able to conclude that reduced planning margins achievable using real‐time tracking of the prostate (3 mm) leads to less radiotherapy‐related morbidity than patients treated with conventional margins. Conversely, a prospective clinical trial with 165 patients treated between 2001 and 2007 found no statistically significant difference in genitourinary toxicity (*p* = 1.0) and gastrointestinal toxicity (*p* = 0.37) between two groups with 10 and 5 mm CTV‐to‐PTV margins^20^ when daily ultrasound‐based image guidance was used. However, potential inaccuracies inherent to ultrasound localization have been demonstrated elsewhere.^21^, ^22^

Given the retrospective studies that indicate reduced planning margins will lead to reduced dose to bladder and rectum which results in less toxicity, and the potential of improvements in QOL with margin reduction, the primary objectives of this study were to prospectively assess 1.) Impact of reducing the PTV margins on cumulative (delivered) dose to OAR and PTV, and 2.) Assess the patient reported QOL changes by reducing the PTV margins.

We found that no significant changes were observed between the standard and margin reduced groups when deviation of the delivered dose to PTV and OARs were considered. This was done by using previously validated deformable dose accumulation techniques to estimate the actual delivered dose and comparing against the planned dose. Therefore, the loss of target coverage and changes to OAR doses resulting from reducing margins from 10/4 mm to 5/4 mm are statistically insignificant for the patient cohorts considered in this study.

To assess QOL, we used validated EPIC‐26 methodology that has been used in major prostate cancer studies^7^, ^23^ and has also been recommended for routine clinical use by TrueNTH Global Registry.^24^ We found that the margin reduced group consistently has improved QOL scores compared to the standard group (i.e., QOL_Margin‐reduced_—QOL_control_ > 0) for three EPIC domains *Urinary Irritative/Obstructive, Bowel, and Sexual*. To determine if these outcomes are clinically meaningful, we utilized the Minimally Important Difference (MID) criteria for EPIC‐26 domains established by Skolarus et al. as endpoints. They utilized distribution‐based and anchor‐based approaches to define essential MID values that demonstrate when changes in symptoms for prostate cancer survivors are clinically relevant.^16^ We found out that for these three domains, the QOL difference between the two groups at end of RT, overall mean in the [0–48] month period, as well as 83% of the time points at 2, 6, 12, 24, 36, and 48 month follow ups, are above MID. This suggests that reducing PTV margins to 5/4 mm leads to a clinically meaningful difference in QOL for these three domains. Since the patients were not receiving hormone therapy, we did not expect to see significant changes in *Hormonal* domain.

When looking at possible correlations between dose to OAR and EPIC domains, the only significant correlation observed were between Urinary Incontinence domain and Bladder dose parameters D_mean_, V_80_, and V_50_. No other dose parameters to bladder or rectum showed a significant correlation to any other EPIC domain. This may be due to the relatively small sample size. Even so, these small differences in margins (and dose effects) still led to important clinically improvements in QOL parameters.

We also observed that hot spots in the bladder and rectum are significantly smaller for the margin‐reduced group, while the dose to these hot spot volumes (D_mean,_ D_max,_ and D_min_) remains similar. This is expected behavior when PTV margins are reduced, as it decreases the PTV overlap with bladder and rectum that are adjacent to the prostate. The PTV/rectum intersection volume shows a moderate correlation to the bowel EPIC domain (R = −0.56). No other significant correlations were observed between hot spots in bladder and rectum, and EPIC domains.

One limitation of this study compared with the previous trial by Sandler et al. which used Calypso for real time localization, is that contrary to Calypso system which enables both localization and tracking of the prostate during treatment, CBCT enables localization of the prostate prior to the start of the treatment only. Therefore, intra‐fraction motion is not accounted for. As a result, PTV margins for this trial were set at 5/4 mm, larger than the 3 mm overall margin Sandler et al. used. Of note, Calypso systems involve an invasive procedure to place the fiducial markers and are not as commonly available in routine clinical practices, while CBCTs are widely used. Therefore, employing CBCT localization to investigate how tight the PTV margins could be made to reduce toxicity to the rectum and the bladder without compromising the target coverage could impact clinical practices on a larger scale. Finally, CBCT has the potential advantage of providing true 3D volumetric information, unlike Calypso which involve matching only a few points in space.

In this study, we used in‐house dose accumulation methods that uses Elastix algorithms and energy‐mass mapping, which we've validated previously.^9^, ^10^ However, intensity‐based DIR algorithms are prone to uncertainties in regions of limited image contrast, especially in cases of large deformations or mass changes.^25^ As such, the dose accumulation results must be considered in light of such uncertainties with DIR algorithms.

We acknowledge other study limitations including a relatively small cohorts of patients that precluded robust statistical analysis of the results. Clinical endpoints for a larger cohort will be presented in an upcoming manuscript. While this trial included only conventional RT patients, results could be relevant for hypofractionation as well. However, for stereotactic body radiotherapy to prostate, MR‐guided may be needed for aggressive margin reduction and resultant reduction of toxicity.^26^ On the other hand, the prospectively collected patient‐reported QOL data between two groups in the study are highly encouraging in favor of margin reduction for CBCT‐guided conventional fractionation.

## 5 CONCLUSIONS

Daily deformable dose accumulation shows reducing the CTV‐to‐PTV margin from 10/6 mm to 5/4 mm does not lead to a significant loss of target coverage or OAR dose changes. QOL results in this prospective trial shows that there is a clinically meaningful difference in QOL for the margin reduced group for three EPIC domains. Confirmation with more patients and longer follow‐up is warranted.

## AUTHOR CONTRIBUTIONS

All authors listed have contributed directly to the intellectual content of the paper in accordance with the authorship criteria of JACMP.

## CONFLICT OF INTEREST STATEMENT

Benjamin Movsas, MD, is reporting research support to the department from Varian Medical Systems, ViewRay Inc, and Philips Healthcare. Indrin J. Chetty, PhD, is reporting research grand support from Varian Medical Systems.
